# *Dictyostelium discoideum* as a Platform to Assess the Cytotoxicity of Marine Algal Extracts: The Case of *Glossophora kunthii*

**DOI:** 10.3390/md23110442

**Published:** 2025-11-17

**Authors:** Sheyla J. Figueroa-Valencia, Marcos Hernández, Grover Castañeta, Ian Pérez, Alejandro Ardiles, Elizabeth Figueroa-Valencia, Teresa Cano de Terrones, Francisco P. Chávez, Carlos Areche

**Affiliations:** 1 Department of Chemistry, Faculty of Sciences, University of Chile, Santiago 8320000, Chile; sfigueroav@unsa.edu.pe (S.J.F.-V.); biomjhp@gmail.com (M.H.); groverneoaxel@gmail.com (G.C.); 2 Unidad de Posgrado, Facultad de Ciencias Naturales y Formales, Universidad Nacional de San Agustín de Arequipa, Avenida Independencia s/n, Arequipa 04001, Peru; elizabethfigval@gmail.com (E.F.-V.); dcanof@unsa.edu.pe (T.C.d.T.); 3 Laboratory of Algal Biotechnology, Centre ALGATECH, Institute of Microbiology of the Czech Academy of Sciences, Novohradská 237—Opatovický mlýn, 379 01 Třeboň, Czech Republic; 4 Systems Microbiology Laboratory, Department of Biology, Faculty of Sciences, University of Chile, Santiago 7800003, Chile; 5 Instituto de Investigaciones Químicas (IIQ), Universidad Mayor de San Andrés (UMSA), Av. Villazón N° 1995, La Paz 0201-0220, Bolivia; 6 Departamento de Biotecnología, Universidad Tecnológica Metropolitana (UTEM), Santiago 8320000, Chile; 7 Departamento de Ciencias Básicas, Facultad de Ciencias, Universidad Santo Tomás, Avenida Iquique 3991, Antofagasta 1240000, Chile; aardiles2@santotomas.cl; 8 Department of Experimental Plant Biology, Faculty of Science, University of South Bohemia, Branišovská 1760, 370 05 České Budějovice, Czech Republic

**Keywords:** amoeba, diterpenes, *Dictyostelium discoideum*, *Glossophora kunthii*, seaweed

## Abstract

The social amoeba *Dictyostelium discoideum* is a versatile biological model widely used in drug discovery and studying cellular stress responses. However, its application for cytotoxicity evaluation of natural products, particularly algal-derived compounds, remains underutilized. In this study, we developed a high-throughput developmental assay in *D. discoideum* to analyze the cytotoxicity of acetone and methanol extracts from the Peruvian seaweed *Glossophora kunthii*. Our results showed that the acetone extract caused a transient delay in the social development of the amoeba. In contrast, the methanol extract exhibited no significant effects, even at high extract concentrations. UHPLC/Orbitrap/ESI/MS/MS analysis tentatively identified ten major compounds, including pachydictyol A and dictyotriol A diacetate. The presence of diterpenes, such as dictyotadiol and pachydictyol A, previously reported to exhibit moderate cytotoxic activity, likely explains the developmental delay observed with the acetone extract. This study highlights the utility of *D. discoideum* as a scalable cytotoxicity screening platform within algal pharmacognosy, facilitating the early identification of non-toxic marine natural products suitable for further biomedical and biotechnological development.

## 1. Introduction

Many compounds derived from natural products, which are produced by plants, microorganisms, and marine organisms, have been used as medicines in their original or semi-synthetic form [1,2]. Chemical structures exhibiting remarkable structural diversity from marine organisms represent one of the main pathways in the search for bioactive compounds with pharmacological properties [3]. For instance, macroalgae are rich in bioactive compounds that could be exploited as functional ingredients for both human and animal health applications. Bioactive compounds such as sulfated polysaccharides, furanones, bromophenols, phlorotannins, and terpenoids have been widely investigated and have demonstrated significant antioxidant, antimicrobial, antiviral, and anticancer properties [4,5,6].

*Glossophora kunthii*, considered the taxonomic synonym of *Dictyota kunthii* [7], is a brown alga (Phaeophyceae) belonging to the family Dictyotaceae. It has a membranous thallus of yellowish-brown or olive-brown color, presenting a darker coloration at the basal region. Its morphology is characterized by dichotomous branching and rounded, generally bifid apices [8]. This alga is distributed in South America (Peru and Chile), Australia, New Zealand, and Southeast Asia [9]. The literature has reported the pharmaceutical and health potential of bioactive compounds present in brown algae [5] and the isolation of various compounds contained in them, including 8β,ll-dihydroxypachydictyol A, dictyoxide, pachydictyol A, dilophol, dictyodial [10,11], 9-epidictyol B [12], xenicane derivative [13], dictyotriol A diacetate [14], and crenulide diterpenes [4]. However, few studies have focused on the structural characterization and chemical profiling of these species. Research on other species in the family Dictyotaceae has identified active compounds with antimalarial, antitubercular [15] and anti-leishmaniasis [16] properties, among others, suggesting promising biotechnological potential for *Glossophora kunthii*. Brown algae, in general, are highly valued for their nutraceutical properties and their commercial use in producing bioactive molecules, as well as in the extraction of alginates, carrageenans, and agar meant for its use in the food industry and other sectors [17,18]. Nonetheless, comprehensive studies integrating chemical profiling with functional biological evaluation remain scarce for South American brown algae, including *G. kunthii*.

Cytotoxicity evaluation is a crucial step in the discovery and validation of active compounds, and it is typically performed using mammalian cell lines. However, these systems present several disadvantages, including high maintenance costs and a significant risk of culture contamination, limiting their applicability in large-scale analyses [19]. For this reason, in this study, we propose the use of the amoeba *Dictyostelium discoideum* for cytotoxicity assays due to its importance for drug evaluation in humans, since this model shares several cellular processes and adjacent homologous genes, with the upper eukaryotic cell [20]. Through studies of social amoeba, *Dictyostelium discoideum* has been widely used as a model organism in cell and developmental biology, providing valuable insight into processes such as cell differentiation, chemotaxis, and signaling pathways [21]. Moreover, this amoeba has also been employed in pharmacological trials, where drugs that affect mammalian cells have shown similar effects in this model, although sometimes at higher concentrations [22].

Therefore, this study proposes the social amoeba *D. discoideum* as both an alternative model and a high-performance platform to perform a cytotoxicity screening of acetonic and methanolic extracts from Peruvian seaweed *G. kunthii*. In addition, the comparison and identification of the chemical profiling of these extracts were performed based on UHPLC/Orbitrap/ESI/MS/MS.

## 2. Results

### 2.1. Properties of Extracts from the Alga Glossophora kunthii

**Figure 1:**
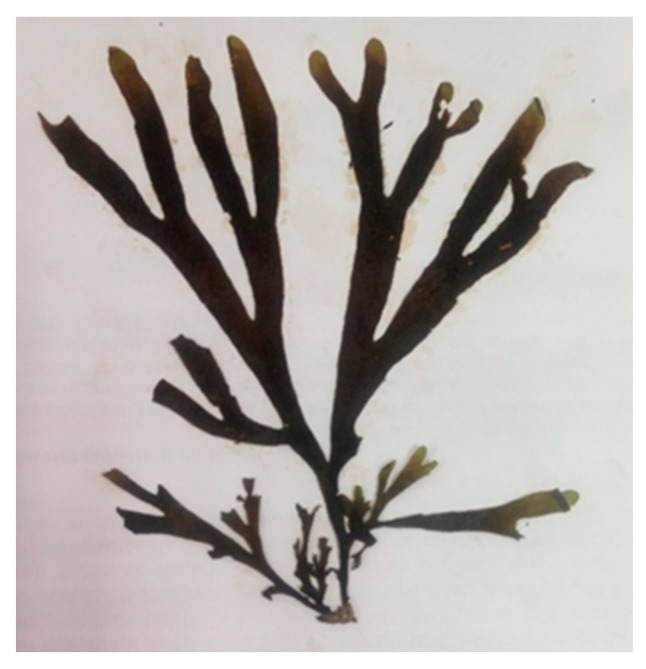
*Glossophora kunthii*.

The extracts from *G. kunthii* (500 g; Figure 1) were obtained using acetone and methanol as solvents through sequential maceration, at room temperature (72 h each). When required, the process was supplemented with direct sonication and centrifugation. This approach yielded 13 g of the acetone extract and 22.5 g of the methanol extraction. Non-polar extracts were not prepared because they primarily contain lipophilic compounds, such as fats and pigments. In this study, we report the isolation and identification of two diterpenoids, known as pachydictyol A [11] and dictyotriol A diacetate, with ^1^H-NMR data identical to those previously reported [14].

Concerning cytotoxicity studies, pachydictyol A has shown some anticancer activities [23], moderate cytotoxic activity on different cell lines (HepG2, WI/38,VERO, MCF-7), and moderate antitumor activity through Ehrlich in vitro assay alongside weak to moderate antioxidant activity for ABTS [24]. It has also presented potent antithrombotic [25] and antiplatelet and anticoagulant activities [26]. Regarding the terpenoid dictyotriol A diacetate, it was isolated from *Dictyota binghamiae* [14] for the first time. There is no literature describing any biological tests for dictyotriol A diacetate.

### 2.2. Social Development Test of Amoeba D. discoideum for Cytotoxicity Assessment

**Figure 2:**
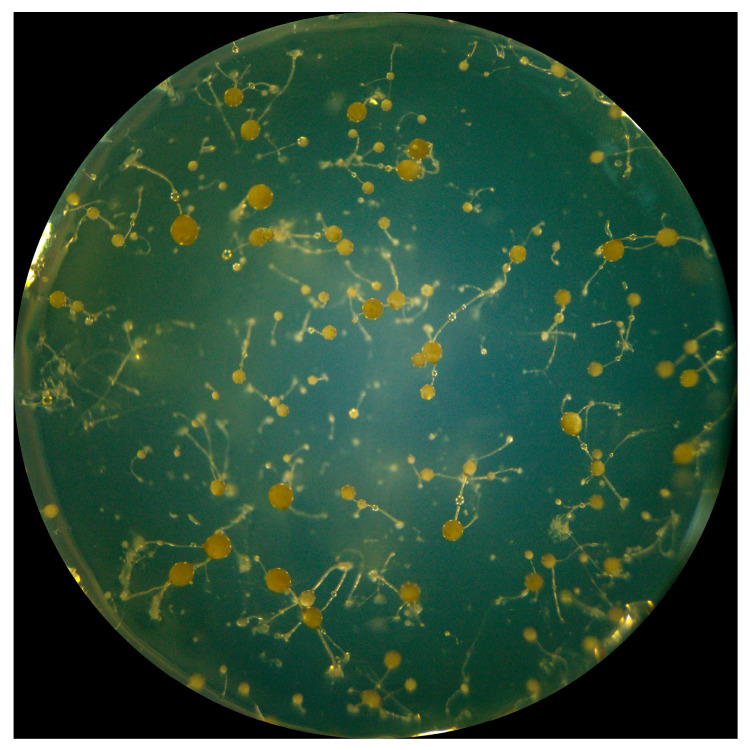
Social development of the amoeba *Dictyostelium discoideum*.

We evaluated the cytotoxic activity of algal extracts by analyzing the social development cycle of the amoeba model *Dictyostelium discoideum* (Figure 2), following protocols previously established in our laboratory [27]. This assay was based on the principle that secondary metabolites, with cytotoxic activity present in the extracts, can inhibit or delay the social development of *D. discoideum*. In contrast, non-cytotoxic compounds allow for the normal progression and completion of their social cycle.

This study evaluated and compared two extracts (acetone and methanol) from the seaweed *G. kunthii*. The social development of the amoebae was monitored over six days, using three concentrations (25, 50, and 100 µg/mL) of each extract. Under normal conditions, *D. discoideum* completed its social cycle in approximately 48 h [28].

**Figure 3:**
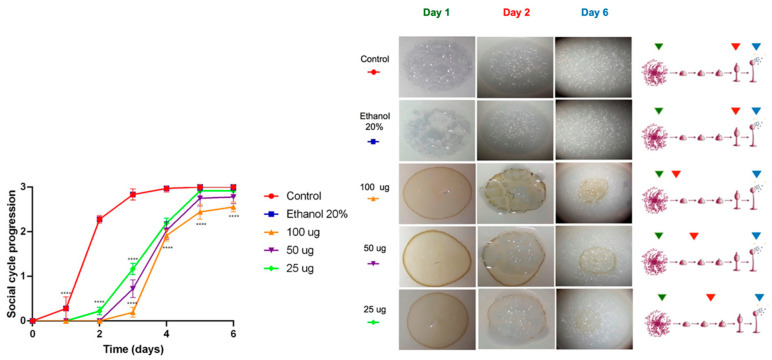
Cytotoxicity evaluation of the acetonic extract of *Ghossophora kunthii*. Statistical analysis was performed using a two-way ANOVA test with multiple comparisons and Dunnet’s post-test (*n* = 6) (* = *p* < 0.05, ** = *p* < 0.005, *** = *p* < 0.001, **** = *p* < 0.0001).

As shown in Figure 3, the acetone extract affected the social development of the *D. discoideum* in a concentration-dependent manner. At all tested concentrations, a significant delay (**** *p* < 0.0001) in the progression of the social cycle was observed, particularly during the first two days, when treated amoebae remained in the early aggregation and phagocytic plaque formation stages, while the control group had already reached the culmination phase. However, by the end of the six-day experimental period, all treated amoebae successfully completed their social cycle, indicating that the effect of the extract was transient and primarily associated with the initial stages of multicellular development.

**Figure 4:**
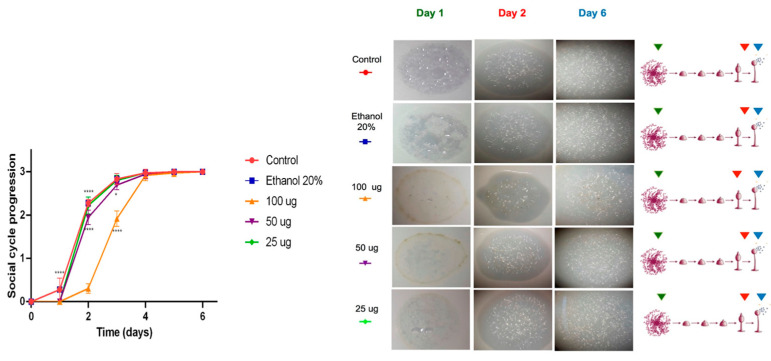
Cytotoxicity test performed by monitoring the social development of *D. Discoideum*. Cytotoxicity evaluation of the methanolic extract of *Ghossophora kunthii*. Statistical analysis was performed using a two-way ANOVA test with Dunnet’s post-test for multiple comparisons (*n* = 6) (* = *p* < 0.05, ** = *p* < 0.005, *** = *p* < 0.001, **** = *p* < 0.0001).

In contrast, the methanolic extracts did not show significant effects on the social development of *D. discoideum*. Phagocytic plaques formed on the first day, similarly to the control group, and only a slight delay was observed at the highest concentration (100 µg/mL) during the first 48 h (**** *p* < 0.0001), as shown in Figure 4. From the third day onward, amoebae treated with all concentrations reached developmental stages comparable to the control, completing the social cycle within the six-day experimental period. Overall, both extracts did not exhibit substantial cytotoxicity; however, the acetone extract caused a moderate delay during the first two days compared with the methanolic extract, which showed a faster recovery in development. These differences were statistically significant only at higher concentration (100 µg/mL), indicating a transient and dose-dependent effect.

Mass spectrometry analysis of the acetone extract from *G. kunthii* identified two diterpenes, pachydictyol A (peak 8) and dictyotriol A diacetate (peak 9), both previously reported to have moderate cytotoxic activity [29,30]. The absence of these compounds in the methanolic extract may account for the lack of significant effects on the social development of the amoebae, suggesting that dictyotriol A diacetate and pachydictyol A are likely responsible for the slight delay observed.

The *Dictyostelium discoideum* model has been shown to be a valuable system for the assessment of the cytotoxic potential of natural extracts. For example, Hernández et al. (2025) [27] employed this model to evaluate dichloromethane and methanolic extracts from various parts of *Helenium aromaticum*. In that study, the methanolic extracts did not exhibit cytotoxicity and allowed the amoebae to develop normally, while the dichloromethane extracts caused developmental delays in a concentration-dependent manner. Additionally, toxicogenomic studies have reinforced the relevance of *D. discoideum* in linking toxic effects, both teratogenic and non-teratogenic, to specific genetic responses [31].

### 2.3. Identification of the Compounds by UHPLC/Orbitrap/ESI/MS/MS of the Extracts of the Peruvian Seaweed G. kunthii

To compare and identify the secondary metabolites present in the methanol and acetone extracts of *G. kunthii*, a combination of diode array detection and high-resolution tandem mass spectrometry (UHPLC/Orbitrap/ESI-MS/MS) was performed. This was performed in positive mode, resulting in the tentative identification of ten compounds, as shown in both Table 1 and Figure S5.

#### Diterpenes

**Figure 5:**
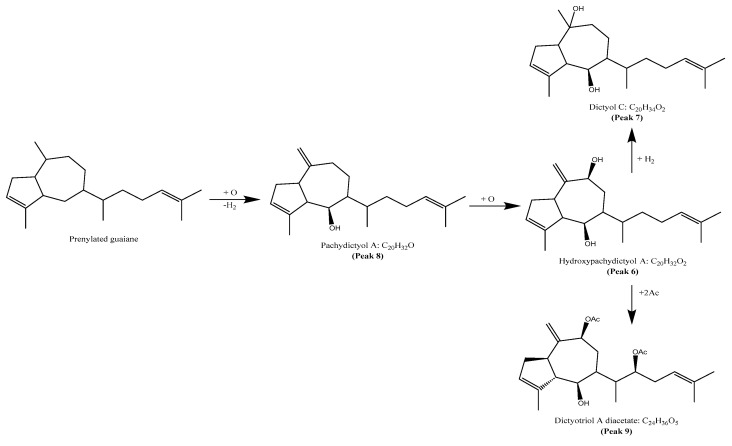
Proposed biosynthetic of terpenes from *Glossophora kunthii*.

The results of the determination of the presence of terpenoids in the acetonic and methanolic extracts revealed a total of 10 compounds, belonging to prenylated-guaiane diterpenes with the perhydroazulene skeleton [32]. They were tentatively identified as pachydictyol A (peak 8, Figure S3) [33], dictyol C (peak 7) [33], hydroxypachydictyol A (peak 6) [34], dictyotriol A diacetate (peak 9, Figure S4) and 4β-acetoxydictyodial A (peak 10) [35]. The biosynthetic proposal [36] of these structures can be seen in Figure 5. Some possible precursors of diterpenes were tentatively identified as patchouliguaiol D (peak 2), showing the molecular ion [MH]^+^ at *m*/*z* 219.1760, two diagnostic ions at *m*/*z* 203.1442 [MH-C_14_H_19_O]^+^, and *m*/*z* 177.1274 [MH-C_12_H_17_O]^+^ [37]. Peak 5 was tentatively identified as hydroxyguaiane sucrose, while peak 1 remained unknown.

Unfortunately, the amount of extract obtained from the algal material was insufficient to allow the isolation and characterization of all these compounds; only two major compounds were isolated and characterized. Future work will be performed toward the isolation and structural elucidation of the remaining compounds.

## 3. Discussion

Marine algae have attracted growing scientific and commercial interest due to their exceptional metabolic capacity to synthesize a broad spectrum of bioactive compounds with diverse biotechnological applications. These metabolites include essential minerals, structural and storage carbohydrates, proteins, polyunsaturated fatty acids, amides, amines, antioxidants, photosynthetic pigments, and an ample repertoire of secondary metabolites with ecological and pharmacological relevance [38,39,40,41,42,43]. Marine macroalgae have emerged as a valuable reservoir for pharmacognosy, which is the study of natural products derived from biological sources with medicinal potential. This places macroalgae at the forefront of marine biotechnology and drug discovery.

Among these ones, brown algae of the genus *Dictyota* stand out due to their widespread distribution in tropical and subtropical marine ecosystems. These habitats are characterized by intense ecological interactions and selective pressures. Such environments have likely driven the evolution of complex chemical defense systems in *Dictyota* species, leading to the production of diverse and structurally intricate secondary metabolites, notably diterpenes. These compounds are known to serve multiple ecological functions: deterring herbivory, preventing microbial colonization, and potentially acting as infochemicals that modulate interactions between symbionts and specialized grazers [44,45].

Interestingly, some primary consumers may have evolved resistance to these deterrents and, in turn, exploit the same compounds for their defense, suggesting a sophisticated form of chemical mediation within the food web.

Diterpenes derived from *Dictyota* are also taxonomically informative, contributing to the chemotaxonomic resolution within the Dictyotaceae family. In this context, the detection of pachydictyol A and dictyotriol A diacetate in *Glossophora kunthii* aligns with prior reports from related taxa [11,14], reinforcing the ecological ubiquity and evolutionary conservation of these metabolites. Their identification not only supports the placement of *G. kunthii* within Dictyotaceae but also highlights the functional consistency of these compounds across phylogenetically related lineages.

Structurally, *Dictyota*-derived diterpenes are typically classified according to the cyclization pattern of their common precursor, geranylgeraniol. The diterpenoids identified in our study, featuring sesquiterpene like ring architectures belong to the prenylated guaiane type class [34,46]. These structures often include core motifs such as the perhydroazulene skeleton and hydroxyl substitutions, which have been linked to pronounced cytotoxic, antimicrobial, and antiviral activities. Minor modifications in stereochemistry, oxidation state, or side chain functionalization can drastically alter the biological properties of these compounds, suggesting that even closely related analogs may exhibit divergent pharmacological profiles. This diversity underscores the relevance of *Dictyota* diterpenes within the scope of marine pharmacognosy, where structure-activity relationships are central to identify new therapeutic leads. Furthermore, our comparison of acetone and methanol extracts reveals the pivotal role of solvent polarity in modulating metabolite recovery. The differential chemical profiles recovered under these conditions underscore the importance of the extraction strategy in natural product discovery workflows.

The action mechanism of structurally related compounds, including pachydictyol C, dictyol E, and 3,4-epoxy-7,18 delabelladiene, isolated from *Dictyota spiralis*, has been previously investigated. These studies demonstrated that, upon exposure to parasitic cells of *Leishmania amazonensis* and *Trypanosoma cruzi*, the mentioned compounds induce a reduction in mitochondrial membrane potential and intracellular ATP levels, chromatin condensation, accumulation of reactive oxygen species (ROS), maintenance of plasma membrane permeability, and marked morphological alterations. Collectively, these observations suggest a cell death mechanism consistent with apoptosis [47]. Moreover, other dictyol-type diterpenes have been reported to inhibit nitric oxide production, suppress inducible nitric oxide synthase (iNOS)m RNA expression, interleukin-6 (IL-6) and cyclooxygenase-2 (COX-2) expression in RAW264 cell [48]. Based on these findings, we propose that the isolated compounds may exert their biological activity through a similar mechanism; however, the present study has not yet elucidated their precise action mode.

Our work highlights the utility of *Dictyostelium discoideum* as a relevant and responsive eukaryotic model for preliminary cytotoxicity screening [22,27]. While this social amoeba does not replicate all aspects of mammalian cell physiology, it offers significant advantages, including genetic tractability, rapid growth, and cost efficiency. Recognized by the National Institutes of Health as a non-mammalian model organism in biomedical research [49]. *D. discoideum* enables early-stage toxicity assessments in a living system. This facilitates the prioritization of candidate compounds for further in vitro and in vivo validation. The observed cytotoxic responses in our assay further support its potential for preclinical screening of marine-derived secondary metabolites. The use of *D. discoideum* as a model organism to assess the toxicity of natural secondary metabolites has been previously described in pharmacological and toxicological studies [27].

Taken together, our findings contribute to the growing body of evidence demonstrating the chemodiversity and bioactivity of Dictyotaceae-derived diterpenes and advocate for the integration of marine chemical ecology, pharmacognosy, and alternative model systems in early-stage drug discovery pipelines.

## 4. Materials and Methods

### 4.1. Preparation of the Seaweed Extracts of Ghossophora kunthii

#### 4.1.1. Seaweed Collection

*Ghossophora kunthii* (C.Agardh) J.Agardh 1882, was collected in December 2017 by Elizabeth Figueroa-Valencia in the Ballenitas and Catarindo, located in the Islay, Arequipa Region, Perú (Figure 1). The sample was deposited with the voucher (N° 0172017) at The Herbario Sur Peruano (HSP)—Michael Owen Dillon Scientific Institute (IMOD), Arequipa, Peru.

#### 4.1.2. Preparation of Algal Extracts

After the cleaning and selection, the seaweeds were dried in an oven at 37 °C. The sample was ground to obtain 500 g of material, and it was subsequently macerated at room temperature for 72 h, this procedure was repeated 3 times. The extraction solvents were filtered and concentrated to dryness by a rotary evaporator at 40 °C.

TLC (Kieselgel 60 GF254, Merck, Santiago, Chile) was developed primarily on *n*-hexane/EtOAc mixtures, and spots were revealed by spraying plates with anisaldehyde/sulfuric acid and heating at 105 °C. Silica gel (Kieselgel 60, Merck, Santiago, Chile, 0.063–0.200 mm) and Sephadex (LH-20, Sigma Aldrich, Santiago, Chile) were used in column chromatography (CC). Chromatotron model 4924 T was used for the isolation of terpenoids. The rotor was coated with a mixture of aluminum oxide 60 GF-254 and calcium sulfate hemi-hemihydrate in thin-layer chromatography; the layer thickness was 2 mm. Technical solvents used in chromatography processes were previously distilled and dried according to standard procedures.

### 4.2. Structure Analysis and Characterization of Isolated Compounds

The compounds were isolated from acetone extract obtained by maceration; the crude extract obtained after solvent evaporation was fractionated by flash column chromatography (SiO_2_). Each fraction was subjected to repeated permeation with Sephadex LH-20 under TLC monitoring. Finally, diterpene mixtures were separated by centrifugal Thing-Layer Chromatography (Chromatotron). The isolated compounds were dissolved in CDCl_3_ for spectroscopic analysis using Bruker Advance (400 MHz for ^1^H). TMS was used as an internal standard.

Compound **1** was obtained as a yellow viscous substance. HR-ESI/MS: *m*/*z* 289.2578 ([M + H]^+^, calc. for C_20_H_33_O 289.2531). ^1^H-NMR (CDCl_3_, 400 MHz): δ 2.67 (q, 1H, J = 10 Hz), 2.51 (m, 1H), 5.33 (br s, 1H), 2.28 (m, 1H), 3.92 (br d, 1H, J = 8 Hz), 1.55 (m), 1.51 (m), 2.63 (m, 1H), 1.25 (m), 2.25 (m), 2.04 m; 1.93 m, 5.09 (br t, 1H, J = 6 Hz), 1.68 (br s, 3H), 1.81 (m, 3H), 4.74 (br s, 1H), 4.72 (s, 1H), 0.96 (d, 3H, J = 7 Hz), 1.65 (br s, 3H). Proton chemical shifts suggest that compound **1** could correspond to an azulene-type diterpene, pachydictyol A, with the molecular formula C_20_H_32_O. This compound has previously been reported in several species of the genus *Dictyota*, such as *D. dichotoma* and *D. menstrualis*. The identification was confirmed by comparing the experimental data with those reported in the literature by Abou-El-Wafa et al. (2013) [50] (Figures S1 and S3).

**Figure 6:**
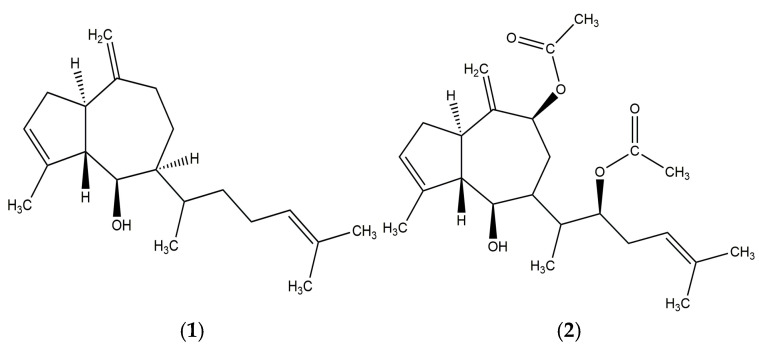
Structures of the two major compounds isolated from the acetone extract of *Glossophora kunthii*: Pachydictyol A (**1**) and Dictyotriol A diacetate (**2**).

Compound **2** was obtained as a white solid. HR-ESI/MS: *m*/*z* 403.2467 ([M + H]^+^, calc. for C_24_H_35_O_5_ 403.2484). The ^1^H-NMR (CDCl3, 400 MHz) spectrum showed δ =2.79 (q, 1H, J = 10 Hz), 2.95 (br s, 1H), 2.49 (m, 1H), 5.34 (br s, 1H), 2.33 (m, 4H), 3.87 (dd, 1H, J = 3, 8 Hz) 3.04, 1H, 2.02 (dd, 1H, J = 3, 10 Hz), 1.92 (ddd, 1H, J = 2, 10, 15 Hz), 1.76 (dd, 1H, J = 6, 15 Hz), δ: 5.59 (dd, 1H, J = 2, 6 Hz), 1.86 (m, 1H), 4.92 (m, 1H), 5.10 (t, 1H, J = 7 Hz), 1.64 (br s, 3H), 1.83 (m, 3H),5.10 (s, 1H), 5.06 (s, 1H), 0.94 (d, 3H, J = 7 Hz), 1.71 (br s, 3H), 2.07 (s, 3H), 2.06 (s, 3H). Compound **2** was identified as the diterpene dictyotriol A diacetate by comparing its NMR data with those reported for *Dictyota binghamiae* by Pathirana et al. (1984) [14] (Figures S2 and S4). Compound **1** and **2** are shown in Figure 6.

### 4.3. Identification by Using UHPLC Q/Orbitrap/ESI/MS/MS

The compounds present in the seaweed *G. kunthii* extracts were tentatively determined using UHPLC Q/Orbitrap/ESI/MS/MS in positive mode. These experiments were based on the previously tests reported by Salgado et al. (2017) [51]. The extracts (1 mg) were prepared with HPLC methanol and then injected into the UHPLC equipment.

The UHPLC system used was a Thermo Scientific Dionex Ultimate 3000 with quaternary RS pump and column oven as part of Thermo Scientific Dionex Ultimate 3000 Series TCC-3000RS, equipped with an autosampler and a high-speed PDA detector using Chromeleon 7.2 software: Thermo Fisher Scientific, Waltham, MA, USA and Dionex Softron GmbH, Bremen, Germany. Analytical chromatography was performed on a UHPLC C18 column (150 mm × 4.6 mm ID, Thermo Fisher Scientific, Bremen, Germany) at a temperature of 25 °C. Four UV detection systems at 254, 280, 320, and 440 nm were used, together with the PDA detector in the 180–800 nm range. The mobile phases were a 1% formic acid solution in water (A) and acetonitrile (B). The elution gradient (time (min), % B) was as follows: (0.00, 5); (5.00, 5); (10.00, 30); (15.00, 30); (20.00, 70); (25.00, 70); (35.00, 5), with 12 min allowed for column equilibration The flow rate was 1.00 mL/min, and the injection volume was 10 μL. The HESI II and Orbitrap focus spectrometer parameters were optimized, as previously reported by Garneau et al. (2013) and Simirgiotis et al. (2016) [52,53]. The hybrid instrument was equipped with quadrupole and orbitrap, with a resolution of 70.000 at *m*/*z* 200) and HCD (high-resolution) cell, using independent variable data acquisition (vDIA) for untargeted analysis. This allowed for the identification of unknown compounds. Daughter ions were produced in the HCD cell at a collision energy of 5 eV, resulting in high sensitivity and accurate mass measurements, with mass errors ranging between 0.1 and 10 ppm. All ions were formed after the heated electrospray probe (HESI II), which are detected in the orbitrap and fragmented in the HCD cell. After detection, the neutral losses were analyzed using the base peak and full TIC spectra with daughter MS2 ions through Thermo Xcalibur 3.1 software.

### 4.4. Dictyostelium Discoideum Strain and Culture Conditions

The strain *D. discoideum* used in this work corresponds to the axenic strain AX4 (DBS0302402) obtained from the Dicty Stock Center (Figure 2) [54]. It was grown according to the protocols described by [28]. Briefly, the amoeba *D. discoideum* was maintained at 23 °C in SM agar (10 g/L glucose, 10 g/L peptone, 1 g/L yeast extract, 1 g/L MgSO_4_* 7H_2_O, 1.9 g/L of KH_2_PO_4_, 0.6 g/L of K_2_HPO_4_, 20 g/L of Bacto agar, pH 6.5) containing a lawn of *K. aerogenes* DBS0305928 (previously grown in LB; 37 °C, 180 rpm, 18 h). Before the tests, amoebas were grown at 23 °C with stirring (180 rpm) in HL5 medium (14 g/L tryptone, 7 g/L yeast extract, 0.35 g/L NaHPO_4_, 1.2 g/L of KH_2_PO_4_ and 14 g/L of glucose, pH 6.3) in the absence of bacteria (axenic culture) and supplemented with ampicillin (Amp) 100 µg/mL and streptomycin (Stp) 300 µg/mL.

The amoebas were harvested in the early exponential phase (1–2 × 10^6^ cells/mL). Viable amoeba cells were determined with Trypan Gibco^®^ blue (Thermo Fisher Scientific) and counted in the Neubauer chamber. Subcultures (S3 to S5) of the amoeba *D. discoideum* in the exponential phase were used for cytotoxicity tests of *Ghossophora kunthii* algal extracts.

### 4.5. Social Development Test of the Amoeba Dictyostelium discoideum for Cytotoxic Evaluation of Algal Extracts

SM agar plates and a lawn of *K. aerogenes* were used to perform social development assays of *D. discoideum*. To assess the cytotoxicity of extracts, 25, 50, and 100 μg doses were tested, following protocols similar to those used in cell line assays [55,56]. A 20% ethanol solution served as the negative control. Ten thousand amoebas in the exponential phase were inoculated [57], and the plates were incubated at 23 °C. The developmental cycle of *D. discoideum* was monitored daily for 6 days, focusing on the aggregation, culmination, and final culmination stages [58]. Scores from 1 to 3 were assigned based on the stage reached [57]. Data were recorded every 24 h and analyzed using GraphPad Prism 6. This assay effectively detects the cytotoxic activity of compounds by evaluating their impact on the viability and cellular differentiation of *D. discoideum*.

## 5. Conclusions

This study introduces a practical and cost effective platform for early cytotoxicity screening of marine algal extracts using the social development assay of *Dictyostelium discoideum*. By leveraging the organism’s sensitivity to chemical perturbations, this approach enables the rapid evaluation of bioactivity under physiologically relevant conditions. The assay proved to be suitable for detecting differential effects among solvent extracts, highlighting its potential to distinguish between cytotoxic and non-cytotoxic samples in a high-throughput format.

Moreover, the chemical characterization of *Glossophora kunthii* extracts led to the successful isolation of two diterpenes, pachydictyol A and dictyotriol A diacetate, alongside the tentative identification of additional metabolites through high-resolution mass spectrometry. These findings underscore the pharmacognostic value of *Dictyotaceae* species and support the integration of ecological screening models with advanced analytical chemistry to accelerate the discovery of marine-derived bioactive compounds.

Altogether, this work contributes a robust methodological framework to marine natural product research and reinforces the utility of non-mammalian systems in the early stages of drug development.

## Figures and Tables

**Table 1:** Tentative identification of compounds contained in the methanol and acetone extracts from *Ghossophora kunthii* algae by UHPLC/Orbitrap/ESI/MS/MS.

Peak	Tentative Identification	Elemental Composition [M + H]^+^	Retention Time (min)	Theoretical Mass (*m*/*z*)	Measured Mass (*m*/*z*)	Accuracy (ppm)	MS Ions (ppm)	Extracts
1	Unknown	C_24_H_36_O	13.25	340.2766	340.2688	22.9	-	acetone
2	Patchouliguaiol D	C_15_H_23_O	21.38	219.1749	219.1760	−5.0	203.1442 177.1274	methanol acetone
3	Oxodecanoic acid	C_10_H_19_O_3_	21.84	187.1334	187.1269	34.7	129.0702 115.0546 103.0545	methanol acetone
4	Guaiane derivative	C_26_H_47_O_12_	22.50	551.3068	551.3092	−4.4	419.2563 257.1932 241.1978	acetone
5	Guaiane derivative	C_25_H_35_O	25.17	351.2688	351.2625	17.9	229.1456 217.1603 309.2124 311.2285 273.2253 269.1941 231.1611 203.1442 177.1274 175.1121	methanol
6	Hydroxypachydictyol A	C_20_H_31_O_2_	26.02	303.2324	303.2383	−19.5	233.1558 219.1398	methanol acetone
7	Dictyol C	C_20_H_33_O_2_	26.61	305.2481	305.2538	−18.7	287.2420 249.1882 235.1714 221.1553 193.1231	acetone
8	Pachydictyol A *	C_20_H_33_O	26.68	289.2531	289.2578	16.2	271.2463 219.1761 203.1219 189.1436	acetone
9	Dictyotriol A diacetate *	C_24_H_35_O_5_	27.57	403.2484	403.2467	4.6	389.2436 347.1794	acetone
10	4β-Acetoxydictyodial A	C_22_H_33_O_4_	28.71	361.2379	361.2317	17.2	301.2225 305.1658 291.1642	methanol acetone

* Authentic compound, confirmed with NMR spectra (Supplementary Material).

## Data Availability

The data presented in this study will be made available on request from corresponding author.

## Supplementary Materials

The following supporting information can be downloaded at: https://www.mdpi.com/article/10.3390/md23110442/s1, Figure S1: ^1^H-NMR spectrum of compound **1** (CDCl_3_, 400 MHz); Figure S2: ^1^H-NMR spectrum of compound **2** (CDCl_3_, 400 MHz); Figure S3: Mass spectra of pachydictyol A (compound **1**); Figure S4: Mass spectra of dictyotriol A diacetate (compound **2**); Figure S5: UHPLC-Q/Orbitrap/ESI/MS/MS chromatograms of the acetonic (green) and methanolic (red) extracts of the alga *Glossophora kunthii*.

## Abbreviations

The following abbreviations are used in this manuscript:

UHPLC	Ultra-High Performance Liquid Chromatography
ESI	Electrospray Ionization
MS	Mass Spectrometry
NMR	Nuclear Magnetic Resonance
HepG2	Human Hepatocellular Carcinoma Cell Line
WI/38	Human diploid lung fibroblast cell line
VERO	African green monkey kidney epithelial cell line
MCF-7	Michigan Cancer Foundation-7
ABTS	2,2′-azino-bis (3-ethylbenzothiazoline-6-sulfonic acid)
ANOVA	Analysis of Variance
TLC	Thin Layer Chromatography
LH-20	Lipophilic Hydroxypropylated (pore size 20)
CC	Column Chromatography
TCC	Thermostatted Column Compartment
RS	Rapid Separation
PDA	Photodiode Array Detector
UV	Ultraviolet
HESI II	Heated Electrospray Ionization (version II)
HCD	Higher-energy Collisional Dissociation
vDIA	Variable Data—Independent Acquisition
TIC	Total Ion Chromatogram
TMS	Tetramethylsilane
SM	Standard Medium
RAW264	Murine macrophage cell line
