# Supplementary material for: Dictyostelium discoideum as a Platform to Assess the Cytotoxicity of Marine Algal Extracts: The Case of Glossophora kunthii
Source: Mar Drugs. 2025 Nov 17;23(11):442. doi: 10.3390/md23110442 (PMC12654238; doi:10.3390/md23110442)
Supplement: Supplementary file 1 [file marinedrugs-23-00442-s001.zip › marinedrugs-3955066-supplementary.pdf]

# Supplementary File

**Sheyla J. Figueroa-Valencia[1,3,4 †], Marcos Hernández [1,2 †], Grover Castañeta [1,6], Ian Pérez [5], Alejandro Ardiles Rivera [7], Elizabeth Figueroa-Valencia [3,4,8], Teresa Cano de Terrones [3], Francisco P. Chávez[2] * and Carlos Areche [1] ***

[1] Department of Chemistry, Faculty of Sciences, University of Chile, Santiago, Chile; areche@uchile.cl (C.A.)

[2] Systems Microbiology Laboratory, Department of Biology, Faculty of Sciences, University of Chile, Santiago, Chile; biomjhp@gmail.com (M.H.); fpchavez@uchile.cl (F.P.C.)

[3] Unidad de Posgrado, Facultad de Ciencias Naturales y Formales, Universidad Nacional de San Agustín de Arequipa, Avenida Independencia s/n, 04001 Arequipa, Perú; sfigueroav@unsa.edu.pe (S.J.F.-V.); dcanof@unsa.edu.pe (T.C.T.)

[4] Laboratory of Algal Biotechnology, Centre ALGATECH, Institute of Microbiology of the Czech Academy of Sciences, Novohradská 237 – Opatovický mlýn, 379 01, Třeboň, Czech Republic.

[5] Departamento de Biotecnología, Universidad Tecnológica Metropolitana, UTEM, Santiago, Chile; ian.perez@usach.cl (I.P.)

[6] Instituto de Investigaciones Químicas (IIQ), Universidad Mayor de San Andrés, (UMSA), Av. Villazón N°1995, La Paz, 0201-0220 Bolivia; groverneoaxel@gmail.com (G.C.)

[7] Departamento de Ciencias Básicas, Facultad de Ciencias, Universidad Santo Tomás, Avenida Iquique 3991, Antofagasta, Chile; aardiles2@santotomas.cl (A.A.R.)

[8] Department of Experimental Plant Biology, Faculty of Science, University of South Bohemia, Branišovská 1760, 370 05 České Budějovice, Czech Republic; elizabethfigval@gmail.com (E.F.-V.)

† SJF-V and M.H. contributed equally to this study.

* Correspondence: areche@uchile.cl (C.A.); fpchavez@uchile.cl (F.P.C).

## Contents

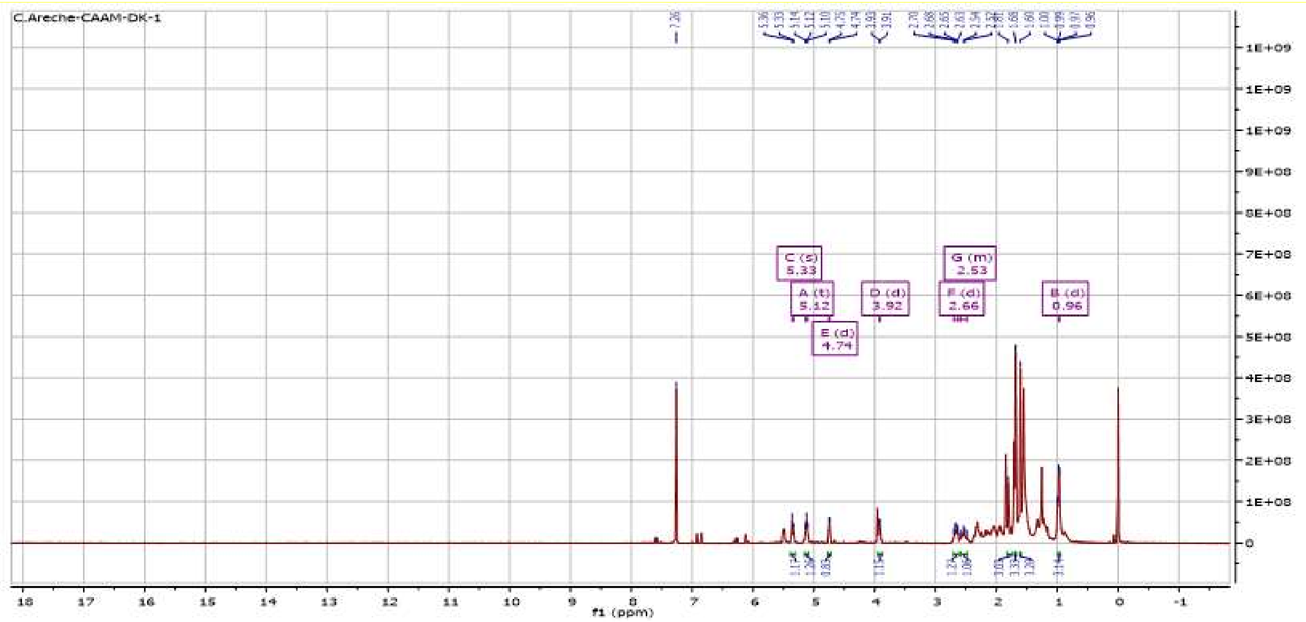


**Figure S1.** $^{1}$H-NMR spectrum of compound **1** ($CDCl_3$, 400 MHz).

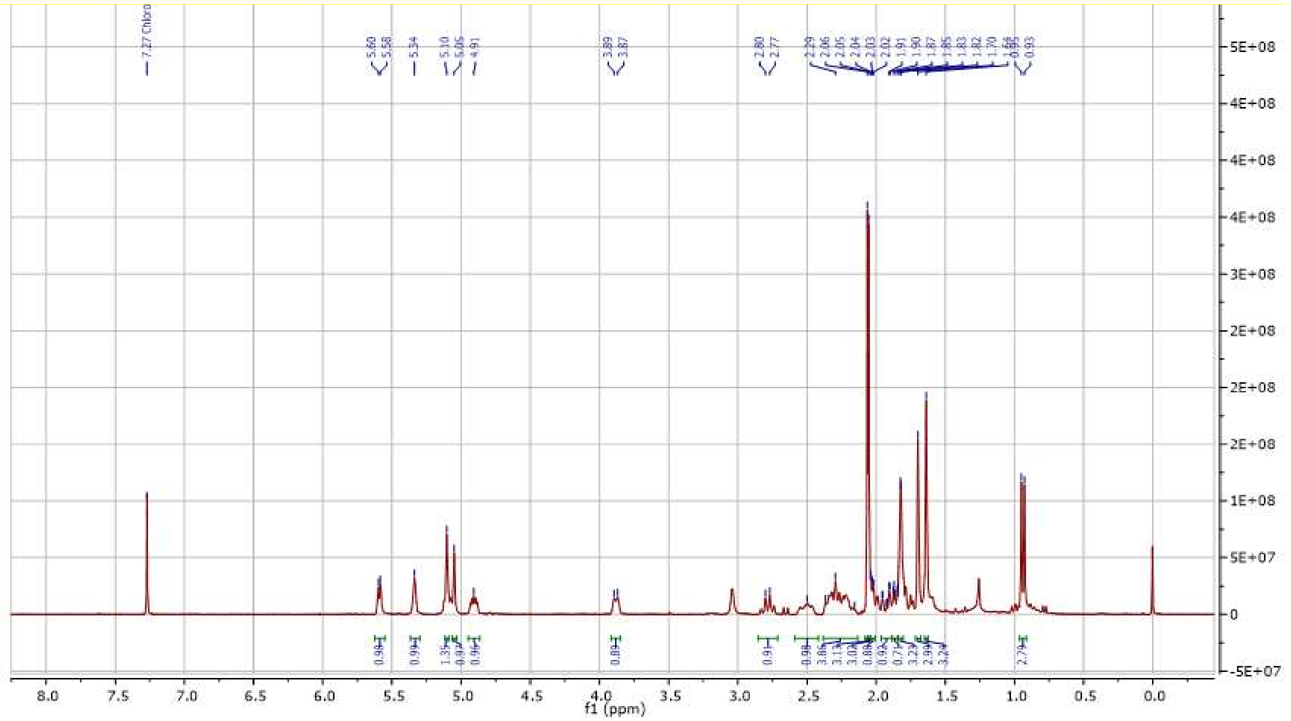


**Figure S2.** $^{1}$H-NMR spectrum of compound **2** ($CDCl_3$, 400 MHz).

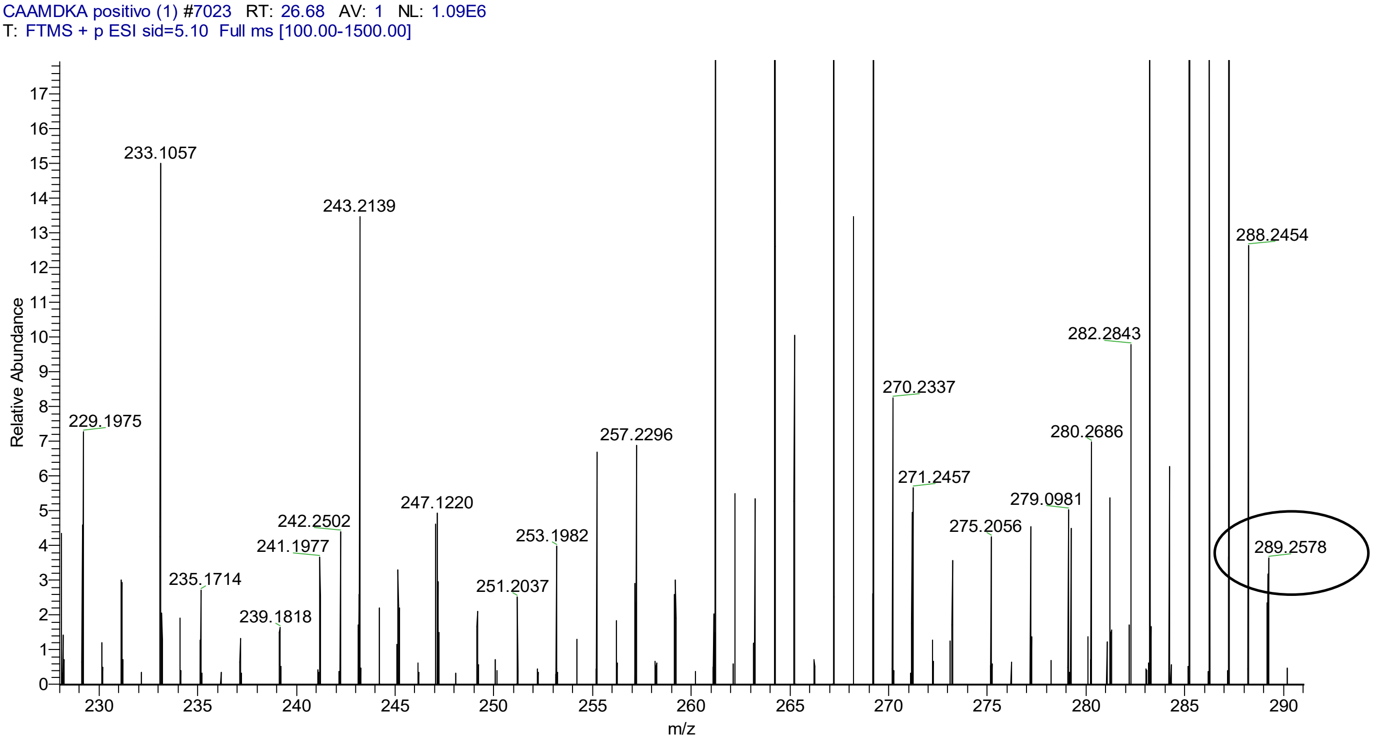


**Figure S3.** Mass spectra of Pachydictyol A (compound **1**).

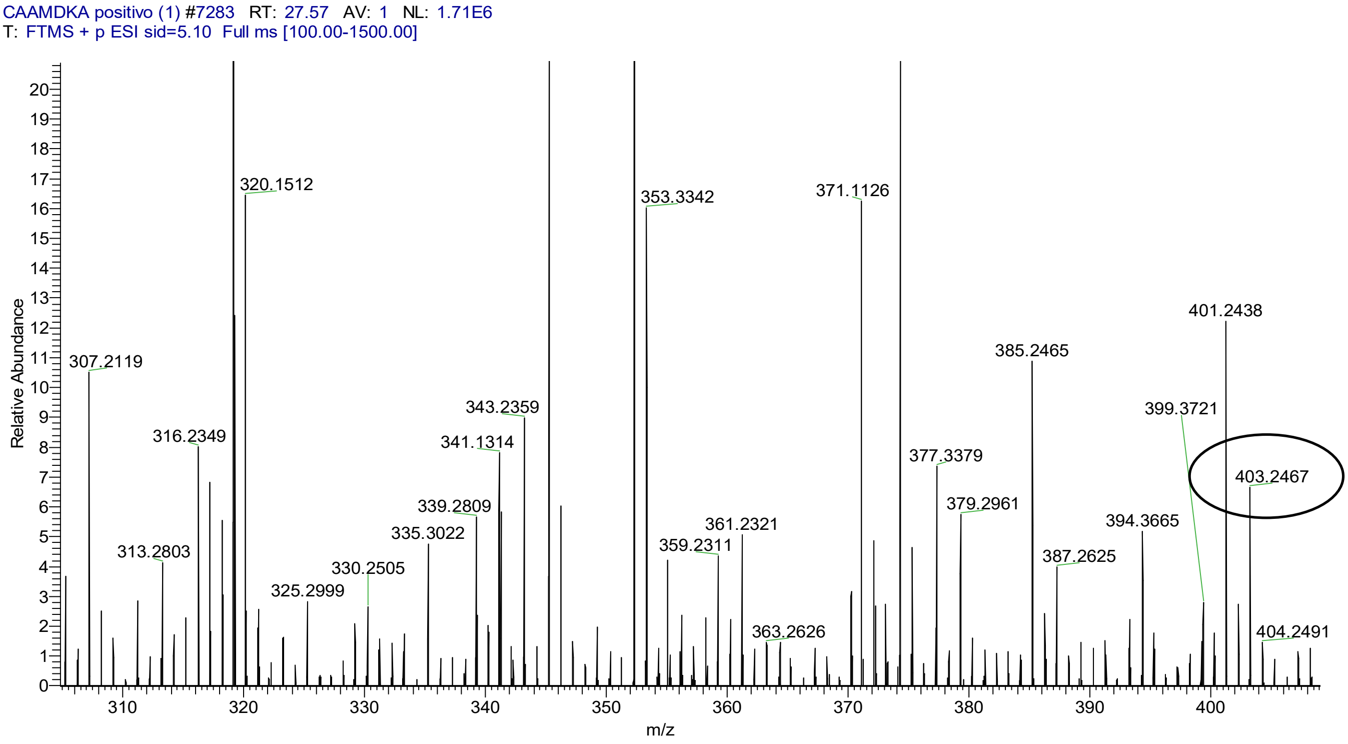


**Figure S4.** Mass spectra of Dictyotriol A diacetate (compound **2**).

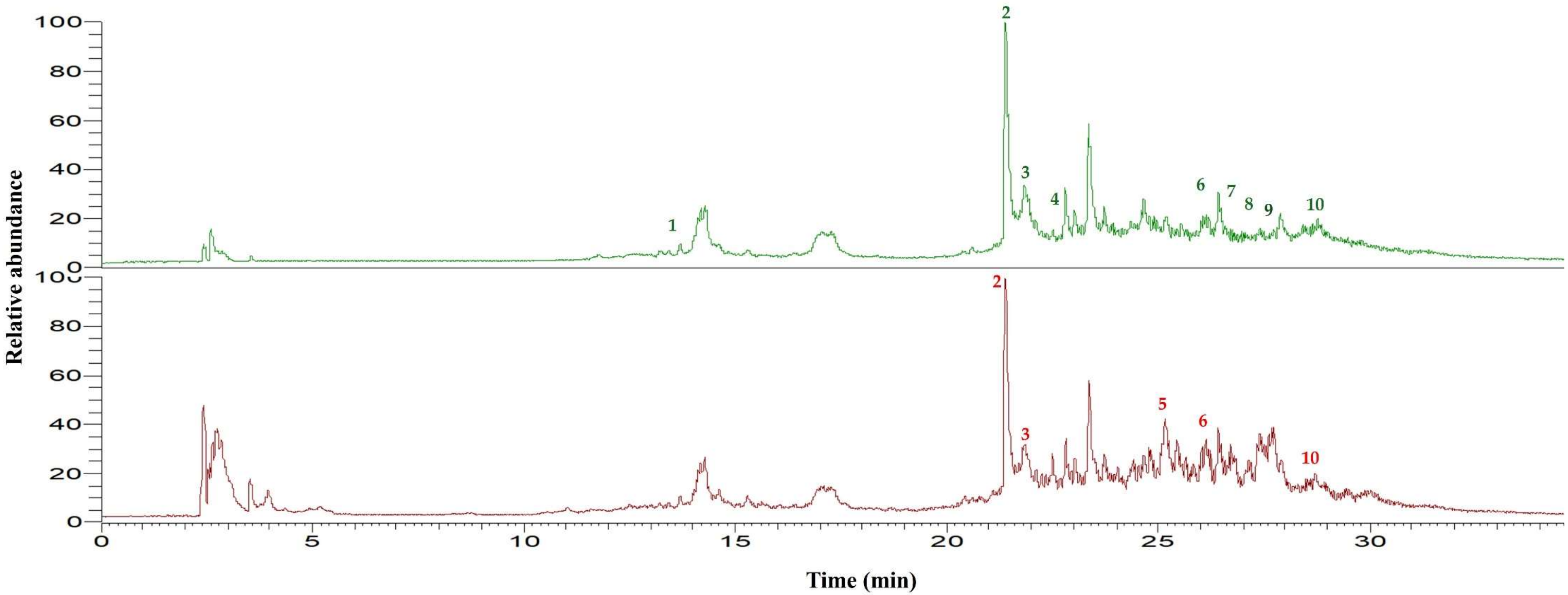


**Figure S5.** UHPLC-Q/Orbitrap/ESI/MS/MS chromatograms of the acetonic (green) and methanolic (red) extracts of the alga *Glossophora kunthii.*
